# BRCA-Monet: a breast cancer specific drug treatment mode-of-action network for treatment effective prediction using large scale microarray database

**DOI:** 10.1186/1752-0509-7-S5-S5

**Published:** 2013-12-09

**Authors:** Chifeng Ma, Hung-I Harry Chen, Mario Flores, Yufei Huang, Yidong Chen

**Affiliations:** 1 Department of Electrical and Computer Engineering, the University of Texas at San Antonio, One UTSA Circle, San Antonio, Texas, USA; 2 Greehey Children Cancer Research Institute, the University of Texas Health Science Center at San Antonio, San Antonio, Texas, USA; 3 Department of Epidemiology and Biostatistics, University of Texas Health Science Center at San Antonio, San Antonio, Texas, USA

**Keywords:** Connectivity Map, Mode of Action (MoA), Breast Cancer Mode of Action Network (BRCA-MoNet)

## Abstract

**Background:**

Connectivity map (cMap) is a recent developed dataset and algorithm for uncovering and understanding the treatment effect of small molecules on different cancer cell lines. It is widely used but there are still remaining challenges for accurate predictions.

**Method:**

Here, we propose BRCA-MoNet, a network of drug mode of action (MoA) specific to breast cancer, which is constructed based on the cMap dataset. A drug signature selection algorithm fitting the characteristic of cMap data, a quality control scheme as well as a novel query algorithm based on BRCA-MoNet are developed for more effective prediction of drug effects.

**Result:**

BRCA-MoNet was applied to three independent data sets obtained from the GEO database: Estrodial treated MCF7 cell line, BMS-754807 treated MCF7 cell line, and a breast cancer patient microarray dataset. In the first case, BRCA-MoNet could identify drug MoAs likely to share same and reverse treatment effect. In the second case, the result demonstrated the potential of BRCA-MoNet to reposition drugs and predict treatment effects for drugs not in cMap data. In the third case, a possible procedure of personalized drug selection is showcased.

**Conclusions:**

The results clearly demonstrated that the proposed BRCA-MoNet approach can provide increased prediction power to cMap and thus will be useful for identification of new therapeutic candidates.

**Website**: The web based application is developed and can be access through the following link http://compgenomics.utsa.edu/BRCAMoNet/

## Background

The ultimate goal of personalized medicine is to design treatments that optimize the therapeutic benefits and minimize the potential risk of toxicity for individual patient. Current pharmacogenomics research is striving to guide compound development and drug selection for such purpose. This growing need for personalized treatment has pushed the development of high-through technologies such as microarray and high throughput sequencing to the research forefront, where compound selection methods based on DNA or mRNA profiling have been developed to achieve highest benefit from therapeutic intervention but at the same time lowest risk of side effects [1-6]. In the meantime, these high-throughput profiling technologies could be applied to elucidate the mechanism of compound treatment in inducing or inhibiting gene expression regulation at different levels. In this study, the focus is on using gene expression profiling for drug screen and effective treatment prediction.

Besides genome-wide association studies, the current gene expression based approaches are mainly based on the "signature gene set" concept, which has been perfected during the past 14 years of relentless efforts in gene expression profiles of cancer, cardiovascular disease, diabetes and other disease researches. [7-10]. The key differences of this "signature gene set" approach from traditional linkage-based genetics study lie in two aspects. First, the "signature gene set" approach can identify genomic variation, being it in SNP, DNA copy number alteration, or miss-regulation of gene expression. Second, it can predict the relevant biological pathways, protein-protein interaction networks, and gene ontology functional groups, thus identifying novel therapeutic targets/biomarkers for drug discovery, with the hope that their variations from patient to patient could explain large portion of dosage variation, resistance and efficacy of the drug [11]. As such, one could also hypothesize that the activities (the relative abundance and interactions) of these signature genes could be part of drug targets, or mode-of-action (MoA), as these genes can be used to explain tumor types and differences in chemotherapeutic response of patients. In other words, activities of signature genes could be used to predict the drug sensitivity. In addition, one may extend this hypothesis further such that this prediction of pharmacological levels in cell type could be extrapolated to other cell types. Applications of these hypotheses have been developed in many studies [12,13]. One of the most notable work is the connectivity map (cMap) project [12], where 4 human cell lines (MCF7/ssMCF7, HL60, PC3, and SKMEL5) were treated by 1,309 chemical compounds at different dosages, and their expression profiles were generated. A prediction algorithm based on gene set enrichment analysis (GSEA) [14] was also developed to rank compounds based on input signature genes obtained from tumor comparison. This project has been widely adapted and developed in the drug discovery area. Several treatment candidates have been discovered for cancer cell lines in the cMap project by directly applying the cMap approach [15-17]. With the idea of searching for 'inverse signature' to the phonotype of interest, this approach has been extended to predict treatment potentials of compounds not included in the cMap project [18-22]. In addition to the original cMap approach, multiple other methods have been developed based on cMap data for new drug repositioning approaches [23-28] or improving the performance of exist cMap [29-31].

Although cMap has been widely applied, problems remain to be resolved for reliable prediction. First, cMap does not differentiate cell lines in its prediction. Often times, the top ranked drugs were from cell lines different from the query cell line. However, our investigation (see Result) suggested that the drug effect is cell line dependent and the higher ranks of the drugs from other cell lines would be more of cell line effects as opposed to drug effects. As a result, considering drug samples from other cell lines introduces only noise to drug prediction. Second, the quality of the data samples in cMap is inconsistent. Some samples from the same drug treatment can behave considerably different from the rest. These samples will inevitably present erroneous predictions. Third, the query signature gene set in cMap is chosen to include the top up- and down- regulated genes. However, size of the gene set is determined quite ad hoc. As a result, one might miss the important signature genes by choosing a smaller gene set, or on the contrary, bring in unrelated genes that would only serve to degrade the prediction. As an example, we used the expression data for estradiol (E2) treated MCF7 cell line [32] as a query to cMap and genes corresponding to the highest 100 and lowest 100 fold changes were used as the query gene set. Naturally, we would expect that E2 ranked high in the predicted list of drugs. However, E2 was only ranked 828 among over 1,200 drugs. The reason for this low ranking is because the result is a summary of the rankings of all cell lines of E2 samples, which are mixed (ssMCF7: rank 12, HL60: rank 31, MCF7: rank 3091, PC3: rank 3508; details in Additional file 1; see also *BRCA-MoNet Application Case 1*). However, even if we focused on E2 for MCF7 cell line, its ranking is still low (3091). Close look at the detailed results revealed that, the ranking E2 treated MCF7 cell line was a summary of the results from 19 individual E2 treated MCF7 cell line and their enrichment scores did not agree with each other (Table 1), Among the 19 samples, only a few have high enrichment scores. It is very likely that the rest of samples do not have high quality and thus fail to catch the real E2 treatment effect. Another potential cause for this poor result is the ineffective choice of the signature genes. However, as a user, we do not have a better way to choose an effective gene set to achieve better prediction. These results underscore the need for quality control and systematic selections of signature genes.

**Table 1:** Detailed prediction result in MCF7 cell line for an E2 treated query sample by the cMap project.

Rank	batch	Cmap name	dose	cell	score	up	down
6	513	estradiol	10 nM	MCF7	.901	.397	-.307
18	725	estradiol	100 nM	MCF7	.800	.244	-.381
39	506	estradiol	100 nM	MCF7	.742	.267	-.314
48	725	estradiol	10 nM	MCF7	.717	.338	-.223
119	757	estradiol	100 nM	MCF7	.629	.367	-.125
489	1010	estradiol	10 nM	MCF7	.432	.179	-.159
731	656	estradiol	10 nM	MCF7	0	.454	.187
738	767	estradiol	10 nM	MCF7	0	.447	.217
741	506	estradiol	10 nM	MCF7	0	.445	.195
745	765	estradiol	10 nM	MCF7	0	.441	.144

To address the above challenges, we proposed BRCA-MoNet in this paper. BRCA-MoNet is advantageous in three aspects compared with cMap. First, it focuses only on breast cancer cell line. Although doing so ignores other cell line data in the cMap data, it nevertheless removes the cell-line dependent interference from the true drug effect. Second, a quality control procedure as well as new drug signature gene set selection algorithm are developed to remove the possible noise in cMap data and characterize drug's treatment effect in a more systematic manner. Third, we define a Mode of Action (MoA) as a group of compounds that share the similar differential gene expression signature. Since the drug expression signature is indicative of the degree of its sensitivity to a cell, a MoA drug group should possess similar therapeutic effect. The construction of MoA introduces extra prediction power. This is because drugs with similar treatment effect might be ranked low due to high noise in data if we treat prediction of each drug independently. In contrast, this high noise sample could be ranked high because the query agrees with its MoA. The MoA is also different from other existing defined compound groups such as those by their anatomical therapeutic compound (ATC) classification since MoA is defined by differential gene expression after treatment, even though some overlapping between various compound classifications might be expected. The relationship of different MoAs in terms of their therapeutic effect can be modeled and visualized by a BRCA-MoNet. BRCA-MoNet presents a global view of drug effects at a genomic level. This network augments and improves the current understanding of compound MoA defined mainly from a physiology perspective, and underscores the relationship of different compounds. From a computational perspective, the MoAs and the quantified relationship between drugs in BRCA-MoNet provide a system-level model crucial for optimal drug screening: a new compound can be easily assigned to a MoA in the BRCA-MoNet such that compound's therapeutic effectiveness can be extrapolated or inferred accordingly.

## Result

### Analysis results showed drug treatment effect is cell line dependent

**Figure 1:**
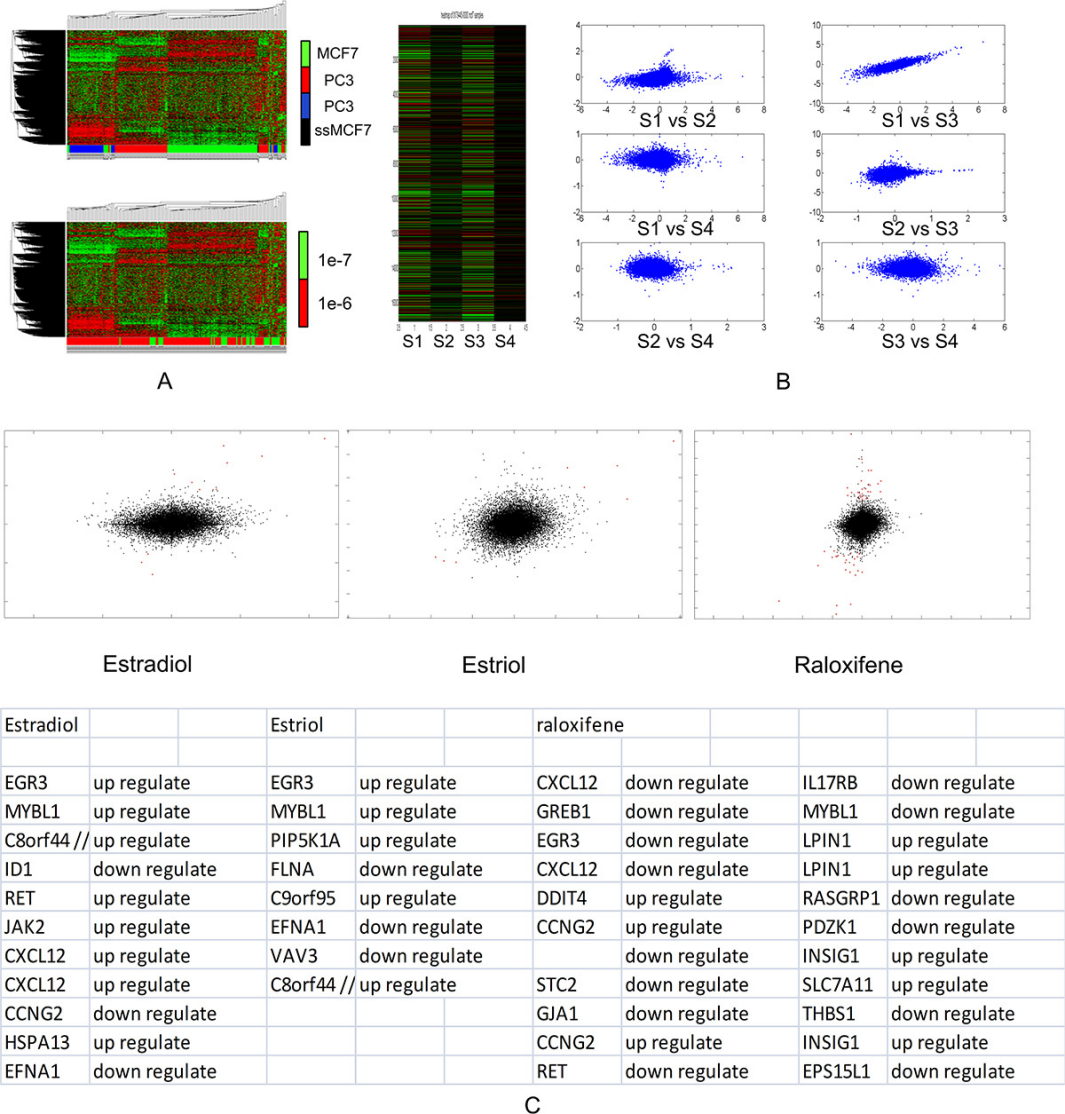
**Result of cell line investigation, signature selection, and quality control**. A) The clustergram of expression samples from (HDAC) families of enzymes. Hierarchical clustering of 175 expression samples treated by drug TRS A. Rows and columns represent genes and samples, respectively. Columns were labeled with cell line (top) or concentrations (down). Clusters can be clearly observed and further examination of samples in the same cluster reveal that they are all from the same cell line. However, no such correspondence presented for the drug concentrations. This suggested that drug effectiveness is cell line dependent. B) Example of Quality Control. The heatmap and pair-wise two-sample scatter-plot of 4 cMap samples from the same drug were shown. They revealed that only two samples showed similarly and the other two did not. In this example, sample s2 and s4 were removed as noise. C) Example of Signature Gene Set Selection. Two-sample scatter-plots of the selected gene signatures for three drugs were plotted. The red cross dots represented the selected genes and the black dots represented the rest of gene. Tables contain the symbols and expression up- or down- regulation for the selected genes of the three drugs.

In the cMap data, each drug treatment profile includes several treated samples from different cell lines. Whether the effects of the same drug treatments differ for different cell lines need to be investigated before a drug MoA network can be constructed. To this end, samples of cMap data were first grouped based on compounds and the compounds with more than 30 samples were retained. Note that since the data have already been normalized and fold changed over the control sample in the same cell line, the cell line dependent bias should be eliminated; any differences in expression levels within the samples of the same compound are manifestation of differences in chemo-effectiveness due to differences in cell line, drug concentrations, or a combination of both. Hierarchical clustering was performed to the samples in each compound group to reveal potential differences in expression patterns within the same compound. Correlating the clustering results with cell line types and concentrations (Figure 1A) revealed that chemo-effectiveness depends mainly on cell lines and is independent of concentration when it is effective. This finding is significant because it suggested that network construction and drug predictions should be performed by considering cell lines separately. Knowing the effect of one drug for treating breast cancer does not provide information on its effectiveness in lung cancer; including samples from cells other than breast cancer cells introduce only noise to drug treatment network construction. As a result, removing samples from other cells mitigates the interference and consequently improves the accuracy and robustness of the prediction result. Since MCF7 breast cancer cell line cohort contains the largest number of samples (2911 compared with HL60 1229 and PC3 1741), and it contains more drug replicate samples than other cell lines, we focused in this work on developing a breast cancer specific MoA network.

### Drug signature gene set selection

The goal of signature gene set selection is to identify a set of genes that have significant differential expression after the drug treatment. However, the use of the conventional differential analysis methods such as *t*-test is hampered by the lack of the biological replicates in the cMap data set. This limitation becomes even severer after the quality control. For the MCF7 cell line, among all 1251 drugs in cMap, only 32 drugs have more than 5 samples and 1055 drugs have ≤ 2 samples. With such small sample size, any statistically based differential analysis becomes infeasible. To this end, we proposed two criteria based on which the signature gene set of drug was selected: first, the signature genes should have high fold-change expression, and second, the fold change levels of the signature genes should be consistently high among the replicate samples. Based on these two criteria, new signature gene set selection algorithm tailored for small samples were developed (see METHOD for details). For MCF7 cell line, among 1251 drugs, signature gene sets of different size were identified for 1108 drugs. No gene sets were produced for the rest 118 drugs because no genes in their samples were consistently differential expressed. There are also 25 drugs which have only 1 sample in MCF7 cell line. As the result, these 118 MCF7 cell line inconsistent drugs as well as the 25 single-sample drugs were removed. Figure 1.C shows the identified signature gene sets for three drugs: Estradiol, estrol and raloxifene. Estradiol (E2) and Estrol are two forms of estrogen, which plays an important role in human breast cancer. It is therefore nature to see that the signature gene sets of these two drugs share many genes that also have similar expression patterns. For instance, genes EGR3, MYBL1 and C8orf33 are significantly up regulated and EFNA1 are down regulated after treated by both drug. Furthermore, these genes are highly relevant to breast cancer. EGR3 encodes a transcriptional regulator that belongs to EGR family and has been shown to be involved in the estrogen signaling pathway in breast cancer [33]. MYBL1 belongs to a group of genes that encode the MYB proto-oncogene protein; MYB has been shown to be highly expressed in ER+ breast tumors and tumor cell lines and is essential for the proliferation of ER+ breast cancer cells [34]. EFNA1 encodes a member of the ephrin (EPH) family. It is highly compartmentalized in normal breast tissue and lost in invasive cancers; it is plausible to observe its down regulation after the E2 treatment. For the third drug, raloxifene, it is a known estrogen receptor modulator aiming at inducing the estrogen level. Our resulted signature includes both EGR3 and MYBL1 genes being down regulated. This similarity between the identified Estrol and Estradiol signature gene sets suggest that they may share similar MoA. In contrast, the reverse correlation between the raloxifene and E2 gene signatures suggest that their MoA may be opposite to each other. Later analysis indeed showed that E2 and Estrol as well as other 15 drugs are detected to be within the same MoA while roloxifene was predicted top ranked in the reverse prediction list with an independent E2 treatment sample (Details in *BRCA-MoNet Application Case 1*). These results demonstrated that the signature gene sets selected by our proposed algorithm are biologically meaningful.

### Quality control

Quality control is applied on the drugs of cMap MCF7 cell line drugs with more than 3 samples. The goal of quality control is to remove the samples that are not consistently expressed with the others. Our investigation of the cMap data revealed that, there was a considerable amount of outlier samples, whose expression patterns differ significantly from the rest in the same drug (Figure 1B). Including these outliers would introduce only noise in defining the MoA and it is therefore important to remove the outlier samples. Note that signature gene set selection could also serve the purpose of quality control since some drugs could end selected no gene set. For MCF7 cell line, as the result of both gene signature selection and quality control, 1564 samples from 747 drugs are identified and removed and 1347 samples from 504 drugs are passed to BRAC-MoNet construction. These samples can be considered to correctly capture the treatment effect on the MCF7 cell line and were therefore used for subsequent investigation.

### Mode-of-Action & BRCA-MoNet generation

**Figure 2:**
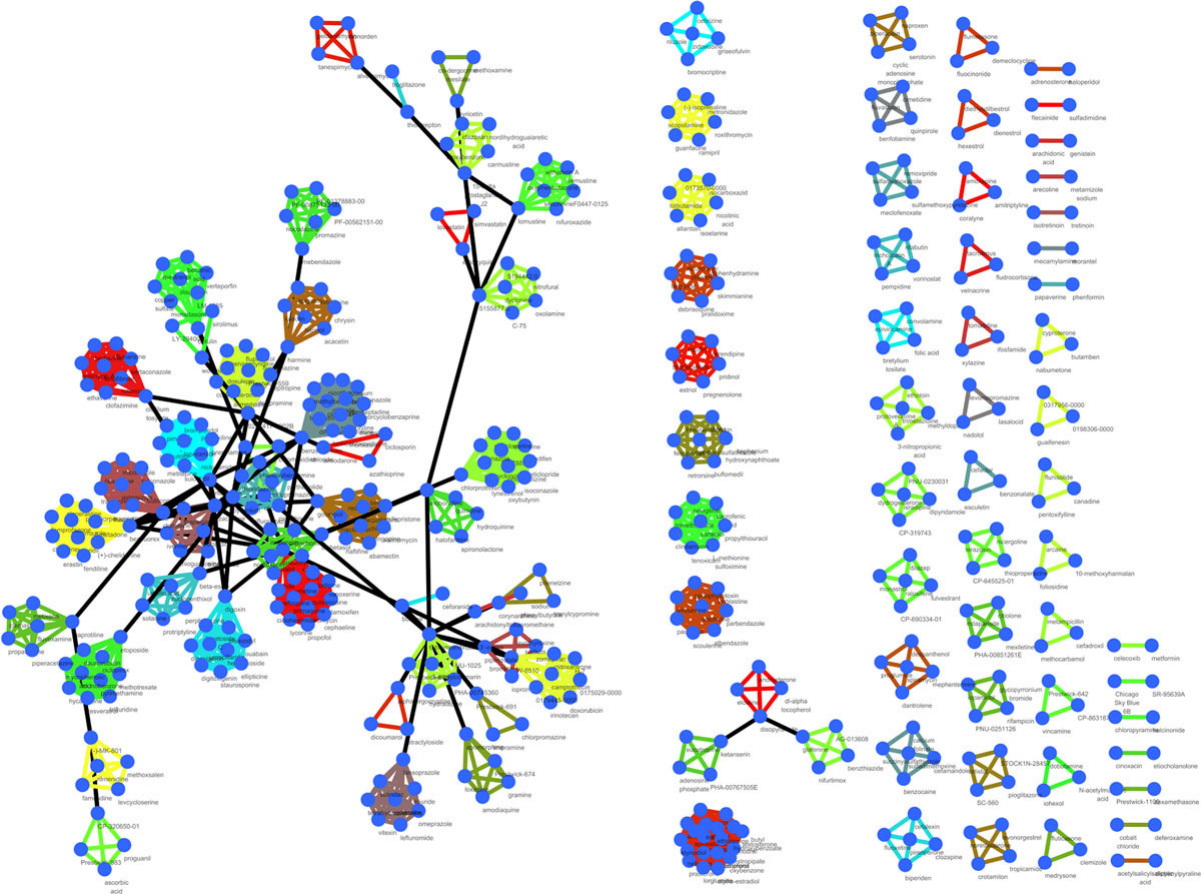
**BRCA-MoNet**. Each node represents a drug. A group of nodes linked by edges of the same color represent a MoA. The black edge linked two MoAs that show correlated effects.

A compound mode of Action (MoA) is defined as a group of compounds that share similar gene signature expression patterns. Drugs forming one MoA will therefore have substantially shared genes in their signature gene set, which also have similar expression patterns. To obtain MoAs, clustering is applied to group the drugs with similar signature gene expression patterns. Multiple clustering algorithms exist and the simple yet effective Hierarchical Clustering (HC) method is adopted in our work. There are two major reasons to choose HC. First, the number of clusters is not required for HC; second, it is reasonable to expect that some drugs form distinct MoAs by itself and HC can produce clusters with a single member. To perform HC, a distance matrix that measures pair-wise distances between drugs was obtained after quality control. With this distance matrix, a total of 109 MoAs were obtained at a threshold and a BRCA-MoNet (Figure 2) was constructed (see *Method *for details). In this network, each node represented one drug; a group of nodes share the same color edges represent a BRCA-MoA obtained by HC. For each MoA, the betweenness centrality of each drug was calculated and the drug with the largest betweenness centrality was set to be the center of the MoA. Two MoAs were linked with a black edge if the distance between them was smaller than the threshold and this link indicated the secondary level relationship between two MoAs.

### BRCA-MoNet application

After the BRCA-MoNet being constructed, its prediction power was tested. Three questions were investigated. First, can BRCA-MoNet predict correct drug MoA? Second, to what extent can BRCA-MoNet predict the drug MoA of an unknown or new drug? Third, to what extend can BRCA-MoNet recommend drugs for patients? To answer these questions, independent microarray expression datasets were downloaded from Gene Expression Omnibus (GEO) for the investigation. In order to avoid possible platform and experimental bias, the following criteria were followed when we select the data sets. First, the data must be compound treated on the MCF7 cell line and contain one or multiple control samples; this was consistent with the condition of the cMap data. Second, we only choose those datasets that were treated with drugs existed in the cMap project or of known treatment effect in breast cancer. Third, to avoid possible across platform complication, the data must be generated from the same platform as the cMap data, or GPL96 (Affymetrix Human Genome U133A Array). With the above considerations, the following three case studies were carried out.

### BRCA-MoNet application case 1: MoA prediction of E2 treated MCF7 cell line & comparison with cMap

We first chose the data set GSE 4025[35] as our query dataset. GSE 4025 includes the MCF7 cell line samples treated with 17beta-estrodiol (E2), a form of estrogen, for 24 hours. We pretended that we do not know the identity of compound (E2) and the goal was to use this treatment sample as a query to our BRCA-MoNet to predict its MoA. Note that E2 is a compound tested in the cMap data and also included in our BRCA-MoNet. Therefore, an accurate prediction algorithm would be expected to rank E2 associated MoA on the top of the predicted MoA list for similar treatment effect and possibly rank MoAs associated with estrogen receptor antagonist at the top of the reverse prediction list. The top similar predictions are shown in Table 2 (See Additional file 2 for the complete result). All the drugs are ranked based on their MoA gene signatures reversely related with E2. In the prediction result, the MoA (BRCA_MoA64) that contains E2 was ranked the 2nd among all the 109 MoAs and E2 is ranked the 4th among the total 504 MCF7 effective drugs selected for BRCA-MoNet. This result indicates that our BRCA-MoNet can achieve very high precision. We investigated more closely the E2 associated BRCA_MoA64 and found that among 17 drugs, 11 are known to be related to estrogen. Specifically, three of them (Estropipate, alpha-estradiol, estrone) were different forms of estrogen and six others (Norethisterone, ethisterone, norethynodrel, levonorgestrel, etynodiol, megestrol) are different forms of progestogen, a precursor of estrogen. Epiandrosterone can induce androgenic activity, which can also lead to a precursor of estrogen, and Epitiostanol is a form of anti-estrogen. Among the remaining six drugs, Naringenin is a weak estrogenic bioflavonoid that exhibits anti-estrogenic activity [32]; Aminophylline is known to interact with estrogen [36]; kaempferol is a dietary flavonoid that functions as a selective estrogen receptor modulator [37-39]; Oxybenzone (also known as benzophenone-3) is a compound widely used in the sunscreen and a few studies suggested that oxybenzone mimics the effects of the estrogen and may cause higher risk to breast cancer; Lorglumide has been shown to induce opposite effect of estrogen in [40]; only nefopam has no evidence that suggests any interaction with estrogen and breast cancer. This significant over-representation of the estrogen related compounds in the E2 associate MoA provides strong evidence to suggest that the constructed MoAs in our BRCA-MoNet do contain drugs of similar effect. Next, we predicted the MoAs with the reverse treatment effect. The result (Table 3; Additional file 3) is equally promising. In the highest ranked MoA (BRCA-MoA 80), two of three drugs (raloxifene, fulvestrant) are selective estrogen receptor modulators, which have anti-estrogenic actions, and the other one (monastrol) is an anti-breast cancer drug [41]. The second ranked MoA, BRCA-MoA86, contains one drug: bacampicilin. Bacampicilin is a penicillin antibiotic and study showed that it interacts with estrogen to reduce the effect of estrogen [42]. The third ranked MoA, BRCA-MoA52, contains two drugs: cyproterone and nabumetone. Cyproerone is a steroidal anti-androgen with additional pro-gestogen and anti-gonadotropic properties. It can suppress the activity of the androgen hormones and subsequently reduce the productivity of estrogen. It has also been studied in clinical I and II trail for its potential as an anti-breast-cancer drug [43].

**Table 2:** Top 20 predicted drugs and corresponding BRC-MoA for similar effect prediction for E2.

Rank	Drug	Mean Expression	MoA
1	0198306-0000	2.060663	BRCA_MoA7
2	guaifenesin	0.136039	BRCA_MoA7
3	0317956-0000	0.127664	BRCA_MoA7
4	Estradiol	2.002982	BRCA_MoA64
5	norethisterone	1.514845	BRCA_MoA64
6	alpha-estradiol	1.459319	BRCA_MoA64
7	estropipate	1.288005	BRCA_MoA64
8	epitiostanol	1.009552	BRCA_MoA64
9	nefopam	0.84901	BRCA_MoA64
10	kaempferol	0.795197	BRCA_MoA64
11	noretynodrel	0.76267	BRCA_MoA64
12	levonorgestrel	0.71241	BRCA_MoA64
13	naringenin	0.554154	BRCA_MoA64
14	aminophylline	0.541906	BRCA_MoA64
15	etynodiol	0.389381	BRCA_MoA64
16	ethisterone	0.239267	BRCA_MoA64
17	epiandrosterone	0.207091	BRCA_MoA64
18	oxybenzone	0.176137	BRCA_MoA64
19	megestrol	-0.40088	BRCA_MoA64
20	estrone	-0.81855	BRCA_MoA64

E2 is ranked 4^th ^(highlighted).

**Table 3:** Top 20 drugs and corresponding BRC-MoA for reverse effect prediction for E2

Rank	Drug	Mean Expression	MoA
1	monastrol	-3.54584	BRCA_MoA80
2	fulvestrant	-1.64228	BRCA_MoA80
3	raloxifene	-1.08114	BRCA_MoA80
4	bacampicillin	-0.39068	BRCA_MoA86
5	cyproterone	-0.95575	BRCA_MoA52
6	nabumetone	-0.32439	BRCA_MoA52
7	proguanil	-0.32213	BRCA_MoA102
8	deferoxamine	-2.16577	BRCA_MoA50
9	cobalt chloride	-0.25959	BRCA_MoA50
10	wortmannin	-0.60183	BRCA_MoA32
11	LY-294002	-0.5634	BRCA_MoA32
12	sirolimus	-0.2227	BRCA_MoA32
13	metampicillin	-0.65666	BRCA_MoA10
14	cefadroxil	-0.25222	BRCA_MoA10
15	methocarbamol	-0.19939	BRCA_MoA10
16	N-acetylmuramic acid	-0.44691	BRCA_MoA33
17	dobutamine	-0.3141	BRCA_MoA33
18	iohexol	-0.15247	BRCA_MoA33
19	adrenosterone	-0.94798	BRCA_MoA58
20	butamben	-0.41198	BRCA_MoA58

This query data were also applied to the original cMap prediction, where the most up- and down-regulated 200 genes were used as the query signature genes. As expected, the cMap project gave a mix results (Table 4) in both predictions of similar-effect drugs (with positive enrichment score) and reverse-effect drugs (with negative enrichment score). E2 itself only ranked 828 (Table 5) in the total 1309 compounds. In cMap, the rank was a summary of a drug's prediction results in every sample of all different cell lines. E2 has a lot of samples in the cMap data across all 5 cell line and the enrichment scores of these samples have large variations, ranging from 0.707 (ssMCF7) to -0.040 (PC3) (Table 6), and this large variation led an insignificant prediction rank. In the reverse effect prediction, Raloxifene, anti-estrogenic modulator, was found to be at rank 9(Table 4) as expected, but fulvestrant, another anti-estrogenic modulator, only ranked 861(Table 7). A closer look at the detailed cell line results revealed that fulvestrant had a negative enrichment score in the MCF7 cell line but a positive enrichment score in the HL60 cell line and the combined result led to a low rank. (Table 8) Over all, the comparison between prediction results of cMap and BRCA-MoNet shows that BRCA-MoNet adds considerable prediction power to the existent cMap data and greatly improves the prediction accuracy on both similar and reverse prediction.

**Table 4:** Top 20 prediction result generated from cMap using GSE4025 E2 treatment signature as a query

Rank	Cmap name	Mean	N	Enrichment	P	Specificity
1	MS-275	-0.954	2	-1.000	0.00000	0.0217
2	Vorinostat	-0.697	12	-0.855	0.00000	0.0266
3	Trichostatin A	-0.597	182	-0.735	0.00000	0.0000
4	Cephaeline	-0.564	5	-0.889	0.00006	0.0361
5	Scriptaid	-0.701	3	-0.962	0.00018	0.0000
6	Prochlorperazine	-0.276	16	-0.506	0.00018	0.0472
7	Mefloquine	-0.481	5	-0.805	0.00064	0.0278
8	5252917	0.657	2	0.977	0.00087	0.0000
9	Raloxifene	-0.465	7	-0.670	0.00114	0.0130
10	perhexiline	-0.476	4	-0.821	0.00193	0.0168
11	Ceforanide	0.199	4	0.813	0.00235	0.0207
12	Bacitracin	0.516	3	0.890	0.00252	0.0068
13	Ikarugamycin	-0.525	3	-0.886	0.00292	0.0152
14	Iloprost	0.353	3	0.876	0.00354	0.0197
15	Isoxican	0.290	5	0.722	0.00375	0.0549
16	Diethylstilbestrol	0.413	6	0.666	0.00399	0.0737
17	Bufexamac	-0.404	4	-0.786	0.00426	0.0000
18	Norcyclobenzaprine	-0.404	4	-0.778	0.00501	0.0310
19	Riboflavin	-0.412	4	-0.777	0.00511	0.0000
20	arachidonyltrifluoromethane	0.576	2	0.946	0.00535	0.0000

**Table 5:** cMap overall prediction result for E2

rank	Cmap name	mean	N	Enrichment	P	Specificity
828	Estradiol	0.209	37	0.367	Null	Null

**Table 6:** cMap detail prediction result for E2

Rank	Name and cell line	Mean	N	Enrichment	P	Specificity
12	Estradiol-ssMCF7	0.700	2	0.989	0.00016	0.0000
31	Estradiol-HL60	0.303	8	0.590	0.00335	0.1585
3091	Estradiol-MCF7	0.222	19	0.485	Null	Null
3580	Estradiol-PC3	-0.040	8	0.223	Null	Null

**Table 7:** cMap overall prediction result for fulvestrant

Rank	Cmap name	Mean	N	Enrichment	P	specificity
861	fulvestrant	-0.295	40	-0.352	Null	Null

**Table 8:** cMap detail prediction result for fulvestrant

Rank	Name and cell line	Mean	N	Enrichment	P	specificity
5	Fulvestrant-MCF7	-0.595	21	-0.714	0.00000	0.0146
180	Fulvestrant-HL60	0.178	6	0.442	0.13728	0.2086
2466	Fulvestrant-ssMCF7	0.000	1	-0.594	Null	Null
3274	Fulvestrant-PC3	-0.032	12	0.413	Null	Null

### BRCA-MoNet Application Case 2: Prediction of BMS-754807 Treated MCF7 Cell Line

One additional dataset treated with drug BMS-754807 was tested against our BRCA-MoNet. This dataset (GSE33366) came from breast xenograft MCF7 bearing mice treated with BMS-754807. MBS-784807 is a dual IGF-1R/InsR inhibitor that can synergize hormonal agents and has been shown to be a potential breast cancer drug [44-47]. Study showed that there is an elevated IGF-IR activity specific in triple negative breast cancer and because of that, BMS-784807 could be a possible treatment for triple negative breast cancer [48]. It has been investigated in several Phase I and Phase II Clinical Trials as an anti-cancer drug [49-52]. This dataset was tested against our BRCA-MoNet for similar treatment effect predictions. The top ranked MoA was MoA 37 (Table 9 and Additional file 4 for complete prediction). Interestingly, this MoA contains valproic acid, which is ranked number 1 among all the 504 BRCA-MoNet drugs. Valproic acid belongs to a general class of drugs called anticonvulsants and was originally used as a non-opioid pain reliever. It has also been used to prevent migraine headaches [53]. Recently, valproic acid has been shown to have great potential as an epigenetic drug for anti-cancer activity through inhibiting cancer cell proliferation in various types of cancer [54-56]. This prediction result shows that both drugs with great anti-cancer potential are actually detected to have similar MoA by BRCA-MoNet. This conclusion strongly supports the fact that BRCA-MoNet can uncover new drug's anti-cancer MoA by assigning it to a known MoA.

**Table 9:** Top 20 drugs for similar effect prediction of BMS-754807

Rank	Drug	Mean Expression	MoA
1	valproic acid	0.159748	BRC_MoA37
2	clobetasol	0.142458	BRC_MoA37
3	labetalol	0.110684	BRC_MoA37
4	tiapride	0.104771	BRC_MoA37
5	cinchonine	0.017354	BRC_MoA37
6	imipenem	-0.00274	BRC_MoA37
7	norfloxacin	-0.09407	BRC_MoA37
8	idazoxan	0.158088	BRC_MoA12
9	nordihydroguaiaretic acid	0.080311	BRC_MoA12
10	carmustine	0.028793	BRC_MoA12
11	15-delta prostaglandin J2	0.021515	BRC_MoA12
12	5155877	-0.0044	BRC_MoA12
13	5194442	-0.01294	BRC_MoA12
14	dioxybenzone	-0.04312	BRC_MoA12
15	C-75	-0.04929	BRC_MoA12
16	(+)-chelidonine	0.15705	BRC_MoA1
17	prochlorperazine	0.032193	BRC_MoA1
18	erastin	0.002357	BRC_MoA1
19	famprofazone	-0.02239	BRC_MoA1
20	clotrimazole	-0.02361	BRC_MoA1

### BRCA-MoNet application case 3: prediction of drugs for UNC breast cancer patients

**Figure 3:**
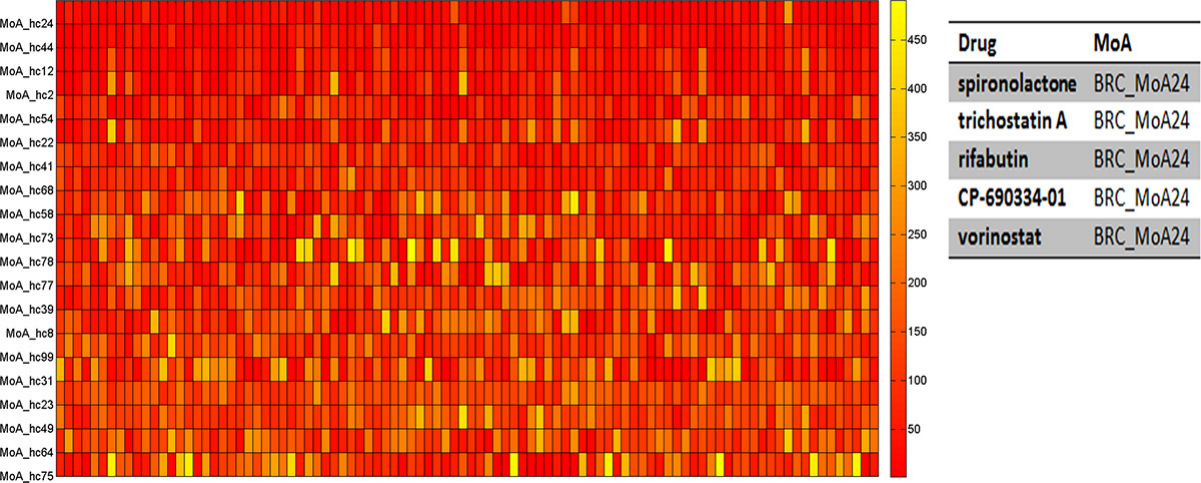
**Prediction result of breast cancer patient**. A) Top MoAs for reverse effect prediction for UNC lumA patients. Color of the heat map indicates the predicted rank of the MoA in an increasing order from red to yellow. B) Drugs of BRC-MoA24.

Prediction power of BRCA-MoNet on the real breast cancer patients was investigated. To this end, dataset GSE2740 [57] was downloaded from GEO. This dataset includes samples from 4 platforms (GPL885, GPL887, GPL1390, and GPL1708) and various breast cancer subtypes. To avoid possible bias due to platforms and breast cancer subtypes, only patient samples of Lumina A (LumA) subtype and from the platform with the largest sample size (GPL1390) were chosen. A total of 97 breast cancer patients' microarray data samples were tested against our BRCA-MoNet using the reverse prediction. The ranking result is shown in Figure 3-A (detailed in additional file 5). Particular, several BRCA-MoAs were consistently ranked at the top, where BRCA-MoA24 ranked the first in 30.21% of the all the patients and ranked above top 20 in 61.46% of all the patients among all 109 BRCA-MoAs. BRCA-MoA24 includes five drugs: spironolactone, rifabutin, vorinostat, trichostatin A and CP-690334-01. Among these five drugs, spironolactone is a synthetic, steroidal anti-mineralocorticoid agent with anti-androgen, weak pro-gestogen properties, and indirect estrogen effects. It has been used to reduce the elevated or unwanted androgen activity in the body [58]. (Androgen, as mentioned before, is the precursor of all estrogens.) So, spironolactone can be potentially used to induce anti-estrogenic activity against breast cancer. Rifabutin is a semisynthetic ansamycin and primarily used in the treatment of tuberculosis. Interestingly, ansamycin has been found to be a HSP90 inhibitor and many of its synthetic compounds are on trials as anti-breast cancer drug. [59-61] Vorinostat is a member of a histone deacetylases (HADC) with a broad spectrum of epigenetic activities; it has been approved by the FDA to treat cutaneous T-cell lymphoma in 2006. Since it has been also shown to have effect on treating breast cancer [62-68], it has undergone multiple Phase I and II clinical trials as an anti breast cancer drug [69-73]. Trichostatin A (TSA) is an organic compound that serves as an antifungal antibiotic and selectively inhibits class I and II mammalian HADC families of enzymes[74]. It has gained extremely high attention in recent years and has been actively studied for its potent antitumor activity against breast cancer ever since 2001 [75-79]. Although the information of the last drug (CP-690334-01) is not available, the overrepresentation of breast cancer related drugs in this MoA gives us a clear vision of the significant detection power of BRCA-MoNet when applied to real patient data.

## Conclusion

A drug effect MoA network for breast cancer cell lines, BRCA-MoNet, was constructed by using the cMap expression data. It was developed to address the problems of the cMap algorithm and to provide robustness and more accurate predictions for treatment effectiveness prediction and drug screening. This improvement came partially as a result of careful quality control on cMap data. In contrast to cMap, BRCA-MoNet prediction is cell line specific and removes the burden for user to select an effect signature gene set. Moreover, BRCA-MoNet assesses the therapeutics influence based on MoA instead of those for individually drugs. This network model not only leads to improved prediction results but it also uncovered the underlying MoA structure of the cMap data that has not been fully discovered before.

The case studies we analyzed here returned favorable results and insightful leads. For the E2 treated MCF7 cell line case, the detection power and insight of the BRCA-MoNet E2-related MoA were exploited. The BMS-754807 case showed that BRCA-MoNet is capable of assigning new anti-cancer drug to the existing anti-cancer MoA and yielding insight understanding of drug MoA detection. The UNC breast cancer patients' case demonstrated the potential of BRCA-MoNet to be used as a tool for personalized treatment recommendation based on patients' gene expression.

The BRCA-MoNet approach provides added values to the connectivity map project and allowed for new and better capability in identification of possible therapeutic candidates. Future direction will likely lend itself to two paths: to expand the MoNet concept to other cancer and cell lines by incorporating multiple drug treatment dataset, and to mature BRCA-MoNet's capability of prediction for the real patients. We expect that the rapid development in cancer profiling projects including The Cancer Genome Atlas (TCGA) will greatly benefit our effort in these future directions

## Method

### BRCA-MoNet workflow

**Figure 4:**
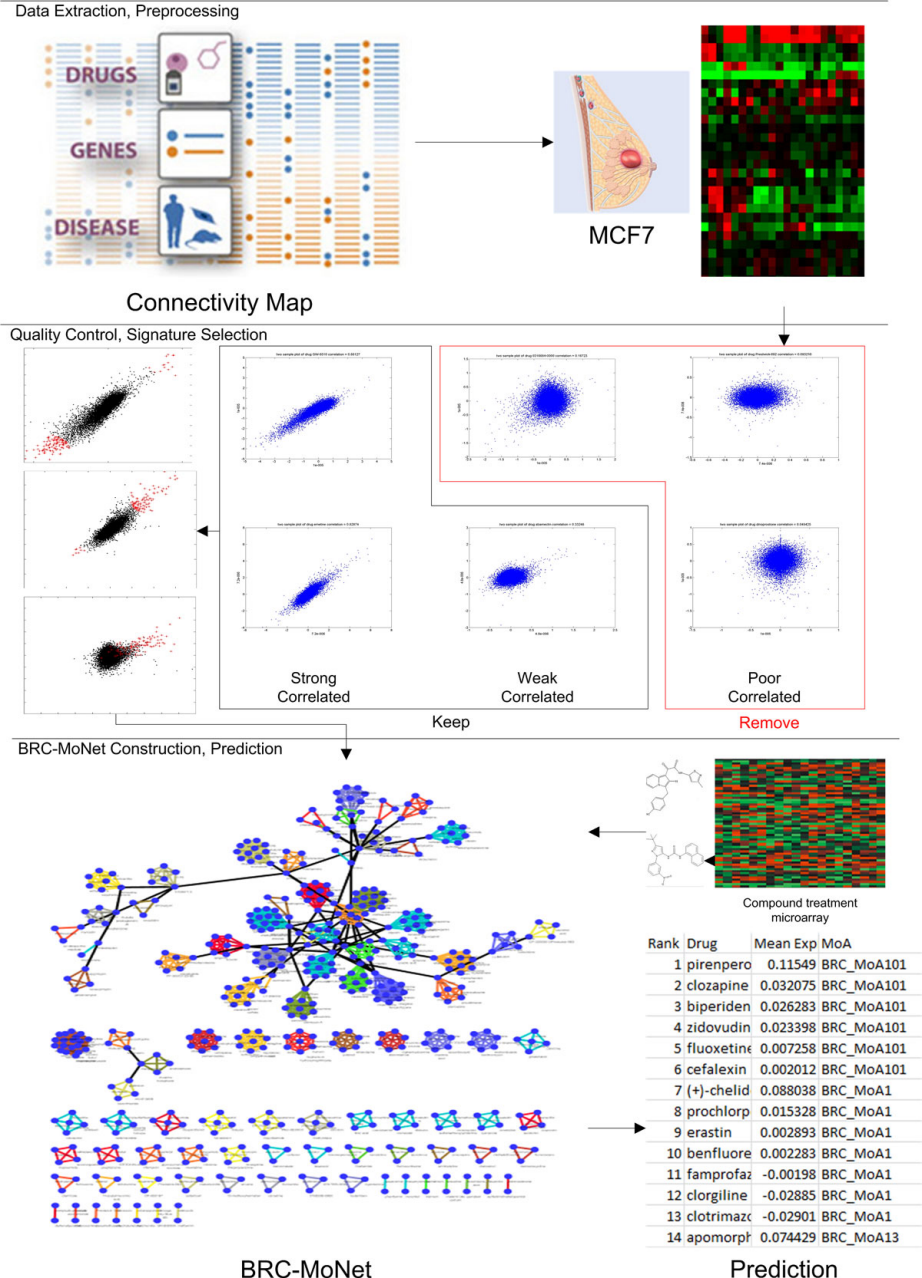
**The workflow of proposed BRCA-MoNet**. The arrow shows the work flow of the project. The whole project can be divided into three parts: 1. Data extraction and preprocessing; 2. Quality Control and signature selection; 3. BRCA-MoNet construction and prediction for new query.

The proposed scheme of generating a breast cancer specific MoA network or BRCA-MoNet from cMap data is summarized in Figure 4. In the first step, new data pre-processing, drug signature selection and clustering algorithms were developed and applied to identify MoAs. In the second step, the relationship between the MoAs in terms of their effectiveness was assessed. Based on the MoAs, the BRCA-MoNet was constructed to depict the relationship of compound effectiveness. BRCA-MoNet and the drug signatures were used for subsequent prediction. Two types of prediction can be carried out with BRCA-MoNet including similar prediction and reverse prediction. For the purpose of find the drug effectiveness on a tumor sample, the expression profile of an individual tumor sample is used as a query, where reverse prediction is adopted and the query will be inverse correlated against the MoAs to predict treatment effects. The prediction result includes a list of MoAs ranked in an increasing order of their negative correlation to the tumor profile. Since effective compounds are expected to have an adverse effect to tumor, MoAs with the negative correlations with the tumor profile will likely be candidates of choice for treating this individual tumor. For the purpose of finding a new compound's treatment effect, a query expression profile from treated sample of a new compound would be used instead as an input to BRCA-MoNet and both similar and reverse prediction results will be of interest as they are the compounds of respective similar and adverse effectiveness in expression. The BRCA-MoNet can be updated when new compound-treated expression profiles are available. One can take the advantage of existing BRCA-MoNet and update it by simply introducing a new MoA and their relationship to other groups. The algorithms are discussed in details in Methods.

### Data preparation

Gene expression profiles of compound treatments were downloaded from Broad Institute's Connectivity Map web site (http://hRp://www.broadinsUtute.org/cmap/). Two Affymetrix arrays were utilized in this study (excluding 184 arrays from early-access version of HT-HG-U133A): HG-U133A (total of 807 arrays) and HT-HG-U133A (6029 arrays), representing 1,267 compound treatments at different dosages. In addition, data includes 5 cell lines: HL60, PC3, SKMEL5 and MCF7/ssMCF7. Each treated sample is accompanied by multiple control/vehicle samples. As for the normalization, the Perfect-Match(PM) probe level intensities, obtained from one Affymetrix array type (including treated and untreated hybridization), was first performed background adjustment together by using Robust Multi-array Average (RMA) procedure. after RMA background adjustment for both array types, quantile normalization was performed to all untreated samples; treated samples were then partitioned according to the array type, vehicle cell-line, and compound; for each group (same array type, cell-line and compound; rank-invariant normalization was performed against their corresponding untreated samples (base line of the normalization was the median of untreated vehicles) at probe-level to correct possible nonlinear abnormality. After normalization, the treated samples expression values were calculated by median polish procedure. At last, all samples (treated and untreated, and both array types) were reassembled into matrix according to Affymetrix probe set IDs.

### Signature gene set selection and distance assessment

The goal of signature gene set selection is to select the genes that are expressed differentially. Since most of the drugs in cMap contains only two samples, the conventional differentially analysis algorithms such as t-test cannot be applied. We proposed the following test statistic to measure if a gene, say *i*, is consistently differentially expressed in a pair of samples

$$R_{i}=\frac{x_{i}}{\sigma_{x}}*\frac{y_{i}}{\sigma_{y}}-\left|\frac{x_{i}}{\sigma_{x}}-\frac{y_{i}}{\sigma_{y}}\right|$$

**Figure 5:**
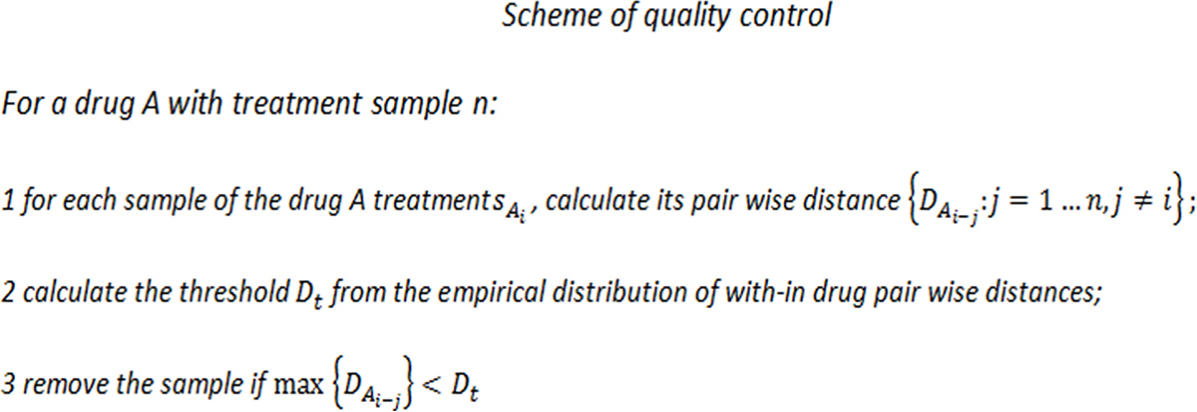
**Pseudo code of the proposed gene set selection scheme**.

Where $x_{i}$ and $y_{i}$ is the expression of gene *i *in sample × and sample y, respectively, and $\sigma_{x}$ and $\sigma_{y}$ are the corresponding sample standard deviation. This statistic values genes which are most differentially expressed in both samples, while taking the sample variation into the consideration. The empirical distribution of this statistic R under the null hypothesis that the gene is not differentially expressed can be obtained by random sampling from replicates of the cMap data. Based on the distribution, p-values can be computed for every gene. A signature gene set of any paired drug samples are determined to contain gene with p-value < 0.1%. The algorithm is summarized in Figure 5. For drugs having a larger sample sized than 2, the procedure of determining signature gene set are fairly the same. Each pair of sample would be used to determine a gene set and then a common subset of all determined gene sets will be the final signature set. Based on the above selected signature gene sets, the distance $D_{ab}$ between any two drug treatment samples *a *and *b *is defined as

$$D_{ab}=D_{max}-\frac{1}{2}*\left(\frac{1}{n}\underset{i^{n}}{\sum }\frac{g_{\left(a-b\right)i}}{var\left(b\right)}+\frac{1}{m}\underset{j^{m}}{\sum }\frac{g_{\left(b-a\right)j}}{var\left(a\right)}\right)$$

where $D_{max}$ is the maximum distance among all pairwise drug treatment samples', $g_{\left(a-b\right)i}$ is the *i*th gene expression level of sample a signature gene set in sample b,n and m are the size of the signature gene sets (the total numbers of genes) for sample a and b, respectfully, and var(a), and var(b) are the sample variance of *a *and *b*, respectfully.

### Quality control

**Figure 6:**
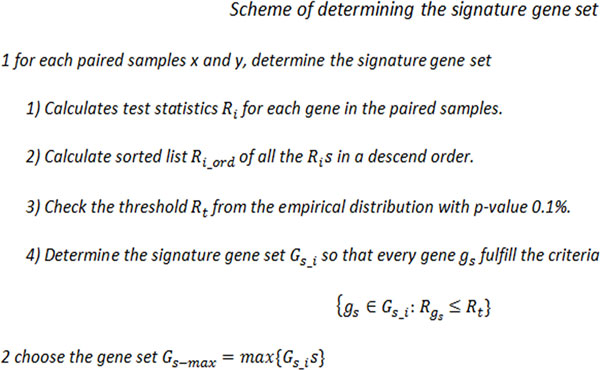
**Pseudo code of the proposed quality control scheme**.

Quality control is done in two rounds of processing. In the first round, which is part of the gene selection, some drugs came by with no signature gene sets; this is a result that no genes were consistently differentially expressed in samples from this drug. The samples from those drugs were removed. Although some drugs were determined with a signature gene set, one or more of the outlier samples may not agree with the rest. To address this problem, a second round of further quality control process was also performed on the cMap samples. In order to remove these inconsistent samples, a new scheme was proposed in Figure 6.

### MoA and MoNet generation

According to the definition of MoA, two compounds are in the same MoA if they share the same genomic signature. This is equivalent to say that the samples from these two compounds are highly correlated. In contrast, the samples from different MoAs should have a correlation distributed according to the distribution of the population correlation. To determine if two drugs *i *and *j *belong to a MoA, a hypothesis testing formulation is developed with the null hypothesis defined by

$$H_{0}:D_{ij}\sim p_{b}\left(D\right)$$

where $D_{ij}$ is the Distance assessment between sample *i *and *j*, and $p_{b}\left(D\right)$ is the the distribution of the population distance. $p_{b}\left(\rho \right)$ is estimated empirically based on the pairwise distances between all sample pairs of the same cell line. Then, a *p *value of 0.01 is chosen as the significance level and the corresponding distance is determined as the threshold. Hierarchical clustering is performed on all the samples distances; then clusters are determined by cutting the linkage at the threshold and the resulted clusters were defined as the MoAs. Notice that since each MoA was generated totally based on the threshold obtained from the background distribution, some MoAs may contain large number of samples while other MoAs only contain few samples from one or two drugs; this is natural and reasonable because some compounds just do not share the treatment effectiveness with others.

Once the MoAs were identified, it was then desirable to reveal the relationship of the MoAs in terms of their therapeutic effects. Instead of investigating individual compound in an isolated fashion, MoNet will enable research to explore a set of compounds (MoAs) that share the same MoA-Signature genes (potential targets), as well as their correlated MoAs.

### Drug Effectiveness Prediction

Using the MoNet and the MoA, one can 1) predict drug effectiveness of a new compound (Similar Prediction) and/or 2) screen compounds to predict the therapeutic effectiveness of different compounds if applied to an individual tumor (Reverse Prediction). For drug effectiveness prediction, the expression profile of cells/tissue treated by a new compound needs to be obtained and the goal is to identify the MoA of the compound. For the therapeutic prediction, a query gene expression profile of the tumor sample is required. The goal is to determine the degree of the adverse relationship between the MoAs and the tumor marker genes expression that reveals how likely the compound is to reverse the expression of tumor marker genes. From the perspective of algorithm development, prediction of drug effect and compound screening are essentially the same. The only difference is the distance criteria: When similar prediction is applied, the MoA is first ranked for the largest positive distance and then each drugs within the MoA are then ranked with the same criteria; when reverse prediction is applied, then the MoA is first ranked for the smallest negative distance and then each drugs within each MoA are ranked the same.

## List of abbreviations used

Connectivity map: cMap; Mode of action: MoA; Breast Cancer Mode of Action Network: BRCA-MoNet; Hierarchical Clustering: HC; Gene Expression Omnibus: GEO; 17beta-estrodiol: E2; The Cancer Genome Atlas: TCGA.

## Competing interests

The authors declare that they have no competing interests.

## Authors' contributions

CM, YH, and YC conceived the idea and designed the experiments. CM and HC prepared the data and conducted the experiments. MF developed the web application. MF, YH and YC wrote the paper.

## Supplementary Material

Additional file 1 **Detailed cMap prediction result for E2 treatment query**. Click here for file

Additional file 2 **Detailed BRCA-MoNet prediction result for similar prediction of individual E2 treatment query**. Click here for file

Additional file 3 **Detailed BRCA-MoNet prediction result for reverse prediction of individual E2 treatment query**. Click here for file

Additional file 4 **Detailed BRCA-MoNet prediction result for similar prediction of individual BMS784807 treatment query**. Click here for file

Additional file 5 **Detailed BRCA-MoNet prediction result for breast cancer patient microarray dataset**. Click here for file

## Declarations

The publication fees were supported by Qatar National Research Fund grant (09-874-3-235).

This article has been published as part of *BMC Systems Biology *Volume 7 Supplement 5, 2013: Selected articles from the International Conference on Intelligent Biology and Medicine (ICIBM 2013): Systems Biology. The full contents of the supplement are available online at http://www.biomedcentral.com/bmcsystbiol/supplements/7/S5.
